# Genome analysis of a coral-associated bacterial consortium highlights complementary hydrocarbon degradation ability and other beneficial mechanisms for the host

**DOI:** 10.1038/s41598-023-38512-z

**Published:** 2023-07-28

**Authors:** Helena Villela, Flúvio Modolon, Júnia Schultz, Nathalia Delgadillo-Ordoñez, Susana Carvalho, Adriana Ururahy Soriano, Raquel Silva Peixoto

**Affiliations:** 1 Red Sea Research Center, Biological and Environmental Science and Engineering Division King, Abdullah University of Science and Technology, Thuwal, 23955 Saudi Arabia; 2 Laboratory of Molecular Microbial Ecology, Institute of Microbiology, Federal University of Rio de Janeiro, Rio de Janeiro, 21941-902 Brazil; 3 Computational Biology Research Center, Biological and Environmental Science and Engineering Division, King Abdullah University of Science and Technology, Thuwal, 23955 Saudi Arabia; 4 PETROBRAS R&D Center (CENPES), Ilha Do Fundão, Rio de Janeiro, 21941-915 Brazil; 5 Marine Science and Bioscience Programs, Biological, Environmental and Engineering Sciences Division, King Abdullah University of Science and Technology, Thuwal, 23955 Saudi Arabia

**Keywords:** Microbiology, Molecular biology, Ecology, Ocean sciences

## Abstract

Here we report the oil degradation genetic potential of six oil-degrading bacteria (ODB), previously used as a bioremediation consortium, isolated from the hydrocoral *Millepora alcicornis* and seawater. The strains were identified as *Halomonas* sp. (LC_1), *Cobetia* sp. (LC_6), *Pseudoalteromonas shioyasakiensis* (LC_2), *Halopseudomonas aestusnigri* (LC_3), *Shewanella algae* (LC_4), and *Brucella intermedia* (LC_5). The taxonomic identification differed from that of the original paper when we used whole genome gene markers instead of just 16S rRNA gene. Genes responsible for the degradation of aromatic hydrocarbons and n-alkanes were found in all genomes, although different (and complementary) steps of the metabolic pathways were unique to each strain. Genes for naphthalene and toluene degradation were found in various strains. We annotated quinate degradation genes in LC_6, while LC_3 and LC_5 presented genes for biosurfactant and rhamnolipid biosynthesis. We also annotated genes related to beneficial mechanisms for corals, such as genes involved in nitrogen and DMSP metabolism, cobalamin biosynthesis and antimicrobial compounds production. Our findings reinforce the importance of using bacterial consortia for bioremediation approaches instead of single strains, due to their complementary genomic arsenals. We also propose a genome-based framework to select complementary ODB that can provide additional benefits to coral health.

## Introduction

Corals, the foundation species of coral reefs, are highly sensitive to oil pollution^1,2^, although they can be even more sensitive to the chemicals used for the mitigation of this toxic compound^2^. In this scenario, bioremediation using oil-degrading bacteria (ODB) has been suggested as a safe and environmentally friendly alternative to protect and restore coral reefs susceptible to oil pollution^1–5^, as previously applied to other organisms and ecosystems^6–8^. In addition, although typically in low abundance in the environment^9,10^, ODB usually respond (and increase in abundance) to the enrichment (e.g., spills) of hydrocarbons^11,12^. When the degradation of excess oil is complete, the oil-degrading bacterial community tends to shift back to its pre-oil community composition^13^. This self-regulating trait is another advantage of biological remediation approaches compared to their chemical counterparts; ODB communities change in relation to the availability of hydrocarbons^12^, whereas chemicals applied to the environment can persist, causing damage to a broad range of organisms^2^. They can also be incorporated into the web chain.

Moreover, although crude oil is essentially formed by a mixture of alkanes, cycloalkanes, aromatics, and heteroatomic compounds containing one or more atoms of nitrogen, sulfur and/or oxygen^14^, the size and complexity of these compounds vary vastly, and different chemical structures have been characterized in petroleum mixtures^15^. Due to the complexity of petroleum-based products, it is unlikely that a unique microbial strain is capable of degrading all oil components^6,16^. Instead, specific microbes harbor different enzymes capable of breaking and utilizing several fractions of the oil as their carbon source^17–19^. Several ODB species have been isolated and used as models to characterize the genes involved in hydrocarbon degradation pathways^20–22^. Studying the potential complementarity of these microorganisms and the beneficial genes that can provide additional protection to the threatened organisms is essential to improve bioremediation efficacy.

Here, we analyzed the genome of six ODB isolated from the hydrocoral *Millepora alcicornis* and surrounding seawater ((*Halomonas* sp. (LC_1), Cobetia sp. (LC_6), *Pseudoalteromonas shioyasakiensis* (LC_2), *Halopseudomonas aestusnigri* (LC_3), *Shewanella algae* (LC_4), and *Brucella intermedia* (LC_5)), collected in Armação dos Búzios, Rio de Janeiro, Brazil^2^. Even though most of the strains were identified to the species level, we will refer to them as *Halomonas* (LC_1), C*obetia* (LC_6), *Pseudoalteromonas* (LC_2), *Halopseudomonas* (LC_3), *Shewanella* (LC_4), and *Brucella* (LC_5) from now on. The consortium composed of these six ODB and fungi was successfully applied in a bioremediation experiment, where its use was correlated with higher crude oil degradation (compared to control samples) and reduced toxic effects on the hydrocoral *Millepora alcicornis*^2^. These strains were therefore classified as Beneficial Microorganisms for Corals (BMCs)^23^ due to their ability to mitigate toxic compounds^24^. In addition to identifying the complementary oil-degrading genomic potential within the bacterial fraction of the consortium, this study also explored other putative BMC characteristics present in each of the six ODB. This in-depth approach allows the prediction of functions that may also be used as part of a “multiple-benefit” oil-degrading bacterial consortium that can also provide other BMC-related benefits, while elucidating the genetic recipe for a complementary and efficient oil-degrading consortium.

## Material and methods

### Bacterial culture and DNA extraction

Six bacterial strains isolated from the hydrocoral *M. alcicornis* and surrounding seawater collected at Praia dos Ossos, Armação dos Búzios, Rio de Janeiro, Brazil (22° 44′ 45″ S, 41° 52′ 54″ W) were originally selected based on their ability to degrade crude oil^2^. The six strains were applied as a consortium in a mesocosm experiment that demonstrated their beneficial effects on coral health and resilience against oil spills. The protocol for the isolation of ODB from corals and reef water is detailed in Villela and collaborators^25^, and the collection site and conditions in Silva and collaborators^2^. The source of isolation for each one of the strains can also be visualized in Table 1.

**Table 1:** General genomic features of each individual strain of the ODB consortium.

Strain	Source	Genome size (Mbp)	GC content (%)	Gene count	tRNA count	CRISPR	Contig count	Optimal Assembly Method	Completeness (%)
LC_1	Seawater	3.63	58.13	3394	56	0	30	Unicycler	100.00
LC_2	Seawater	4.69	41.19	4251	94	0	56	Unicycler	99.95
LC_3	Hydrocoral	3.96	60.71	3713	63	0	70	SPAdes	100.00
LC_4	Hydrocoral	5.01	53.04	4509	112	1	70	CDHit	99.73
LC_5	Hydrocoral	4.57	57.85	4417	51	0	27	SPAdes	100.00
LC_6	Hydrocoral	4.04	62.46	3402	74	0	108	SPAdes	99.14

Briefly, for isolates from the hydrocoral sample, 5 g of sample were macerated in 45 mL of sterile saline, poured into a 125-mL culture flask containing 10–15 units of sterile 4.5 mm glass spheres and kept under constant agitation (150 rcf) for 16 h at 26 ºC. The bacterial isolation was performed by plating 100 µL of each tenfold dilution from 10^−1^ to 10^−6^ in Bushnell-Haas medium supplemented with 1% crude oil as the only carbon source. For seawater samples, dilutions were also performed and plated straight on the plates with crude oil. The plates were incubated at 26 ºC for 3 days. To guarantee the purity of the isolates, colonies were transferred to new individual growth plates with Marine Agar medium (HiMedia Laboratories, LLC, India, using the depletion technique. Individual colonies were grown in Marine Broth (HiMedia Laboratories, LLC, India) under constant agitation (150 rcf) at 26 ºC for 16 h of growth; subsequently, an aliquot was stored in 20% (v/v) glycerol at -80 ºC until use.

### Genomic DNA extraction, sequencing, and assembly

For genomic DNA extraction, 10 uL of the 20% glycerol stocks were freshly streaked in Marine Agar plates. Individual colonies were then inoculated in 5 mL Marine Broth (HiMedia Laboratories, LLC, India) and placed under constant agitation (150 rcf) at 26 ºC for 16 h for growth. From this fresh culture, another plate was streaked to guarantee the purity of the culture, while 1 mL of culture was aliquoted in 1.5 mL conic tubes. The bacterial culture was subsequently centrifuged at maximum speed for 2 min, and the pellet was used for DNA extraction. Genomic DNA extraction was performed using the Wizard Genomic DNA Purification kit (Promega, San Luis Obispo, California, USA) following the manufacturer’s instructions.

A NEBNext Ultra II FS DNA library kit (New England Biolabs, USA) was used to prepare a paired-end 150-bp library of 450-bp, following manufacturer's instructions. The whole genome sequencing of the six ODBs was performed in the Illumina HiSeq 2500 platform. FastQC^26^ and Adapter Removal^27^ software were used for quality assessment and to trim the reads. Subsequently, the trimmed reads were de novo assembled using SPAdes v.3.10.0^28^, Edena^29^ and Unicycler^30^, and then CD-HIT package^31^ was used to remove the redundant contigs, producing a final contigs file. The optimal assembly was chosen based on representativeness criteria, considering assemblies with sizes similar to the expected value for further analysis. The quality assessment of each assembly was checked in CheckM^32^. Default parameters were used for all software.

### Phylogenetic analysis

The JSpecies database^33^ was used to search for genomes related to the studied strains using the tetranucleotide signature correlation index (TSC). Subsequently, the genomes with higher similarity were compared using Average Nucleotide Identity (ANI). The closest species were then selected for phylogenetic correlation using the Multi-Locus Sequence Analysis (MLSA) approach, which is widely used for higher resolution of the phylogenetic relationships of species within a genus or genera within a family^34^. Briefly, we selected and extracted different housekeeping genes (genes that encode proteins with conserved functions), which can be used as marker genes to determine the phylogenetic relationship among the bacterial strains (Supplementary Table S1). The reference genomes were downloaded from the NCBI RefSeq database. The genes were then extracted and concatenated in the same order shown in Supplementary Table S1. The gene sequences were aligned using Multiple Alignment using the Fast Fourier Transform (MAFFT) tool. The phylogenetic trees (Maximum Likelihood, with 500 replicates for bootstrap) were then generated from the evolutionary model previously defined from the lowest BIC score (Bayesian Information Criterion), using the MEGA X software^35^. Genome-to-Genome Distance Calculator 3.0^36^ was used to analyze the digital DNA-DNA hybridization (dDDH) and evaluate the similarity between the studied strains and the closest reference genomes.

### Gene prediction and functional annotation

Genome annotation was performed using Prokka software^37^ and Rapid Annotation Subsystems Technology (RAST)^38^. The search for annotated genes related to hydrocarbon-biodegradation pathways, biosurfactant biosynthesis, ectoine transporters, and dimethylsulfoniopropionate (DMSP) metabolism was carried out manually based on Prokka outputs, searching for the respective gene and protein standard names. RAST results were used to infer functions at subsystem levels. The search for biosynthetic gene clusters (BGCs) was performed on the web-based platform antiSMASH 5.0^39^ and reinforced on PRISM 4 software^40^. Putative symbiotic islands (pSIs) were predicted using GIPSy (Genomic Island Prediction Software)^41^ using default parameters. Reference genomes used as subjects were downloaded from NCBI RefSeq. The ID codes of each reference genome used in GIPSy were: GCF_900129255.1 (query for *Halomonas* sp., LC_1), GCF_001641615.1 (query for *Pseudoalteromonas* sp., LC_2), GCF_002197985.1 (query for *Halopseudomonas* sp., LC_3), GCF_001598875.1 (query for *Shewanella* sp., LC_4), GCF_900454225.1 (query for *Brucella* sp.*,* LC_5), and GCF_000754225.1 (query for *Cobetia* sp.*,* LC_6).

### Pangenome analysis

To identify genomes suitable for pangenomic analysis, we downloaded high-quality genomes from NCBI using the Genome search tool and applied several filters, including a year of release between 2010 and 2023, assembly level of scaffold to complete, and exclusion of atypical genomes. Using these filtered genomes as references, we performed pangenomic analysis for each isolate in our study (queries) using Anvi’o version 7.1^42^ to identify and recover singleton genes. Singleton genes are defined as genes present in only one isolate in comparison to the reference genomes of the same species. We then compared the singleton genes found in each isolate to identify shared and unique genes and assess their unique potential to benefit to the host.

## Results

### Genomic general features and whole-genome-based phylogeny

**Figure 1:**
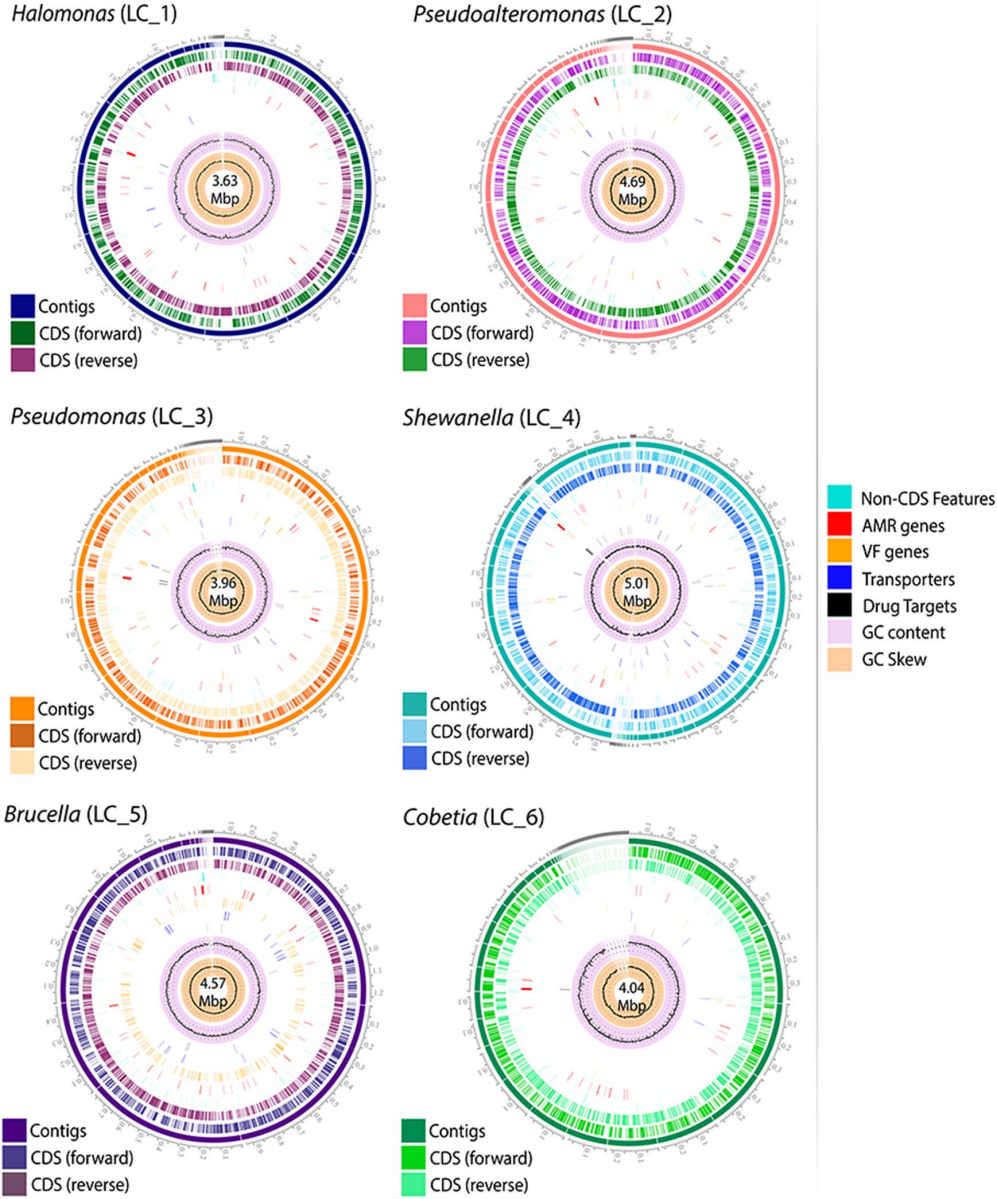
Genomic maps with general features of each individual strain of the ODB consortium. The outermost circles in each of the six genomic maps represent the contigs. Moving inwards, the next circle has the forward CDS, and the following circle has the reverse CDS. Inside each map, the genome sizes are shown.

The genome assembly of the six ODB strains resulted in a total of 27 to 108 contigs per strain. The genome assemblages of all six strains showed sizes ranging from 3.63 to 5.01 Mbp. General genomic features are summarized in Table 1 and genomic maps in Fig. 1.

Taxonomic information and species-level classification are summarized in Table 2. The bootstrap value of each strain and the respective reference genome with the best match was 100% for all phylogenetic relationships. These results indicate strong phylogenetic correlations between the queries and reference genomes of their related species (See Supplementary Fig. 1 for the phylogenetic trees. < Phylogenetic trees.pdf > .). The Digital DNA-DNA hybridization (dDDH), as well as % in GC differences and the ANI values, reinforce these results. However, LC_1 showed dDDH values < 70% and above 1% in the differences of GC content in comparison to *Halomonas meridiana* (GCF_900129255.1), indicating *Halomonas* (LC_1) as a putative new species. Although *Cobetia* (LC_6) showed 0.02% of difference in the GC content with *Cobetia amphilecti*, the dDDH was < 70%, also indicating a potential new species.

**Table 2:** Species-level classification inferred by MLSA method.

Strain	Class	Order	Family	Genus/species	dDDH (%)	% in GC difference	ANI (%)
LC_1	Gammaproteobacteria	Oceanospirillales	Halomonadaceae	*Halomonas meridiana*	33.10	1.17	86.31
LC_2	Gammaproteobacteria	Alteromonadales	Pseudoalteromonadaceae	*Pseudoalteromonas shioyasakiensis*	71.50	0.09	95.13
LC_3	Gammaproteobacteria	Pseudomonadales	Pseudomonadaceae	*Halopseudomonas aestusnigri (*= *Pseudomonas aestusnigri)*	73.10	0.23	96.52
LC_4	Gammaproteobacteria	Alteromonadales	Shewanellaceae	*Shewanella algae*	84.80	0.02	98.02
LC_5	Alphaproteobacteria	Rhizobiales	Brucellaceae	*Brucella intermedia* (= *Ochrobactrum intermedium*)	86.10	0.11	98.23
LC_6	Gammaproteobacteria	Oceanospirillales	Halomonadaceae	*Cobetia amphilecti*	59.50	0.02	94.57

Digital DNA-DNA hybridization (dDDH), as well as % in GC differences and average nucleotide identity (ANI), were measured for the closest type strain from the respective ODB in the phylogenetic analysis. Values > 70% in dDDH indicate the genomes belong to the same species, whilst values > 79% indicate the same subspecies. Differences > 1% in GC content suggest distinct species.

### Oil degradation potential: genes related to the degradation of different oil compounds

**Figure 2:**
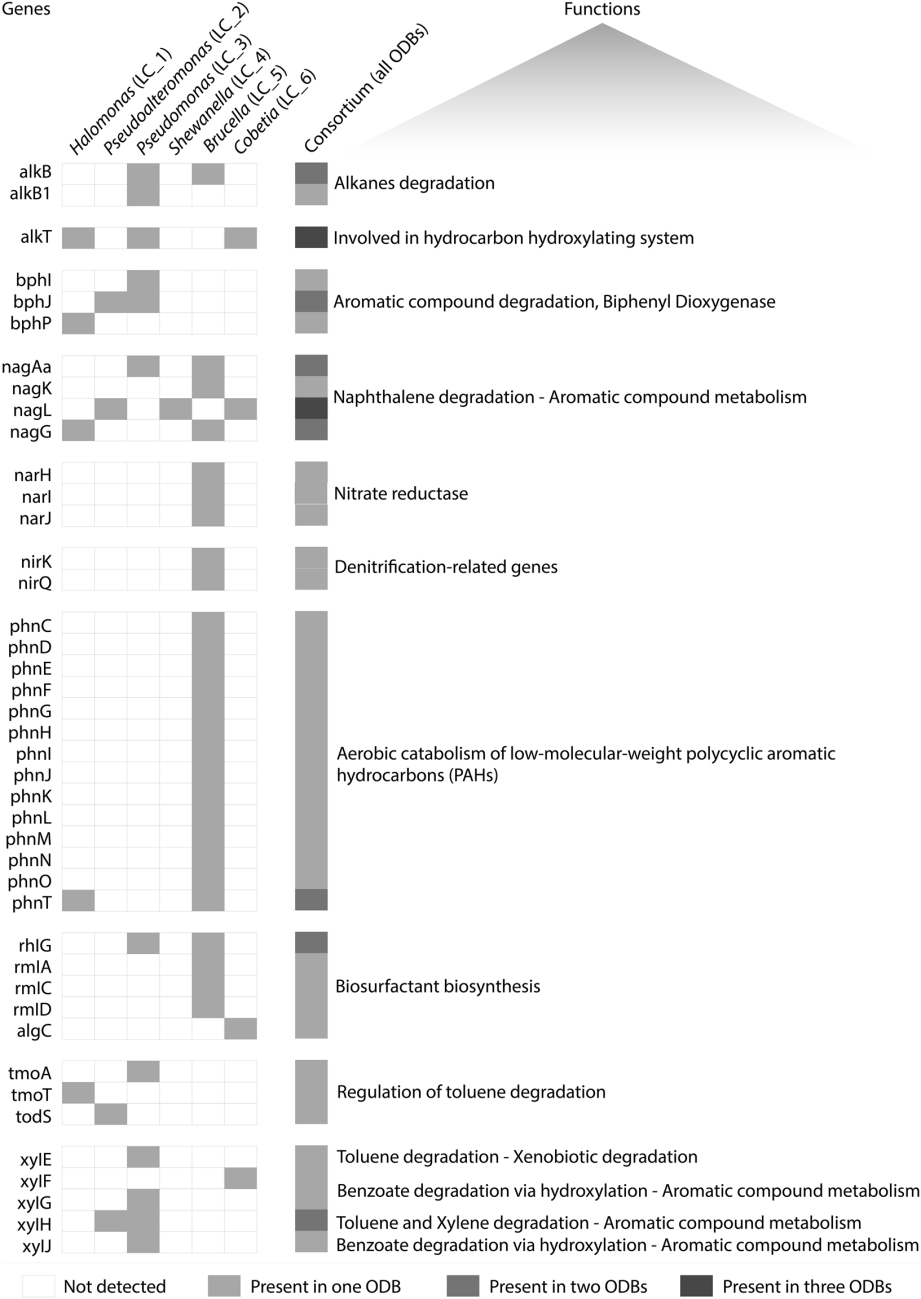
Genes related to hydrocarbon degradation. Grey boxes indicate the presence of the gene on each genome, while blank boxes indicate absence. The last column displays the presence of the genes in the combined consortium.

Eight degradation pathways of aromatic compounds were found among the six genomes analyzed (Fig. 2). We observed a greater abundance of genes related to the degradation of n-phenylalkanoic acid in the *Halopseudomonas* (LC_3), which also showed the highest number of genes related to biphenyl degradation (Fig. 2). Genes related to the metabolism of central aromatic intermediates were widespread across all six strains, as were pathways for gentisate degradation. On the other hand, aromatic amine catabolism-related genes were only annotated in *Brucella* (LC_5). The predominance of pathways related to the Protocatechuate branch of beta-ketoadipate and to the Catechol branch of beta-ketoadipate was observed in *Halomonas* (LC_1) and *Pseudoalteromonas* (LC_2) genomes. *Halopseudomonas* (LC_3) presented the highest number of genes of peripheral pathways for the catabolism of aromatic compounds. In contrast, only one gene was related to the metabolism of central aromatic intermediates—salicylate and gentisate catabolism.

In total, 43 different genes related to hydrocarbon degradation were detected in the genomes of the six ODB strains (Fig. 2). Genes related to naphthalene degradation were found across all the six genomes. *Shewanella* (LC_4) genome analysis revealed only one gene related to hydrocarbon degradation – *nag*L – that is involved in naphthalene degradation. Toluene degrading (*xyl*) genes were found in *Pseudoalteromonas* (LC_2), *Cobetia* (LC_6), and *Halopseudomonas* (LC_3). Alkane degradation (non-cyclic hydrocarbons) genes were identified in *Halopseudomonas* (LC_3) and *Brucella* (LC_5). The strain *Cobetia* (LC_6) presented five different *qui* genes involved in the quinate degradation pathway.

Genes related to biosurfactant synthesis were also investigated. Several genes for biosurfactant production were observed in *Halopseudomonas* (LC_3), and one was detected in *Brucella* (LC_5) and in *Cobetia* sp. (LC_6; Fig. 2). In these same genomes, genes that encode the key enzymes involved in rhamnolipid biosynthesis, a glycolipid biosurfactant, were also found. Genes potentially involved in the biosynthesis of dTDP-L-rhamnose (rhamnolipid) were detected, including algC, mlA, rmlC, and rmlD (Fig. 2).

### Beneficial mechanisms for corals and host-microbial interaction genes

**Figure 3:**
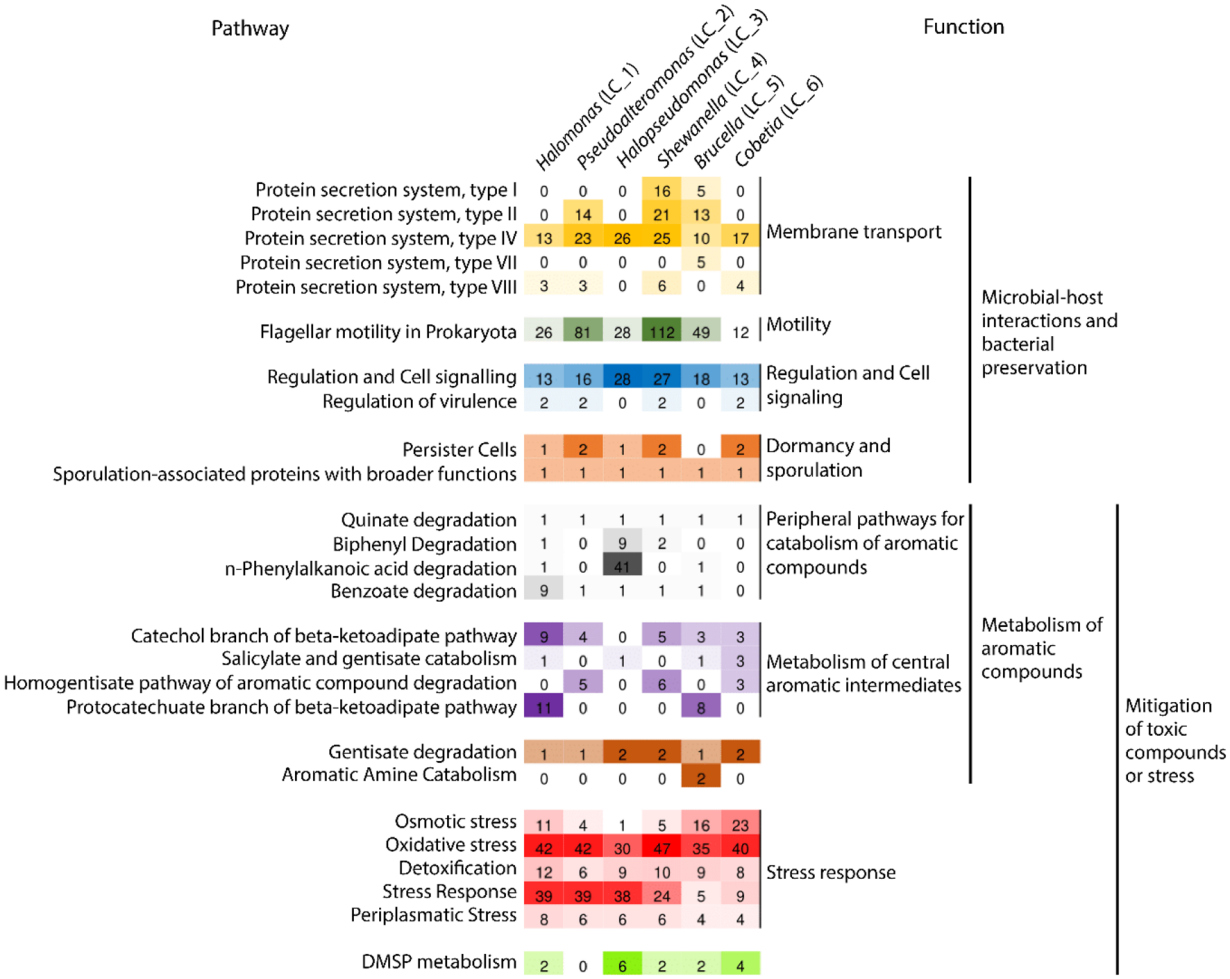
Annotated genes related to microbial-host interactions and/or putative beneficial characteristics for corals detected in the six oil-degrading bacteria studied.

Protein secretion system types I, II, IV, VII, and VIII were detected in the genome annotations (Fig. 3). These are related to transmembrane transport, which has broad functional roles in microbial-host interactions. Type IV secretion system (T4SS) was found in all six genomes, while T8SS was present in four genomes: *Halomonas* (LC_1), *Pseudoalteromonas* (LC_2), *Shewanella* (LC_4), and *Cobetia* (LC_6). T2SS was observed in *Pseudoalteromonas* (LC_2), *Shewanella* (LC_4), and *Brucella* (LC_5), and we also identified T1SS in *Shewanella* (LC_4) and *Brucella* (LC_5). T7SS was only observed in *Brucella* (LC_5).

Putative symbiotic islands (pSIs), symbiotic islands carrying gene clusters acquired by horizontal transfer that play a key role in symbiotic relationships, were annotated in *Halomonas* (LC_1), *Pseudoalteromonas* (LC_2), *Halopseudomonas* (LC_3), *Shewanella* (LC_4), and *Cobetia* (LC_6). Among the pSIs, several genes related to transmembrane transporters were annotated (Supplementary Table S2 < GIPSy-Genes_results.xlsx >).

Aiming to identify other putative beneficial characteristics in these genomes, gene clusters related to specialized metabolites were also investigated. Among all strains, we observed the presence of 28 BGCs, encoding several putative specialized molecules: betalactone, RiPP-like (ribosomally synthesized and post-translationally modified peptides), arylpolyene, ectoine, siderophore, type I polyketides, rare-earth-elements-containing cluster, redox-cofactor, polyunsaturated fatty acid cluster/heterocyst glycolipid synthase-like PKS (PUFA,hglE-KS), terpene, N-acetylglutaminylglutamine amide (NAGGN) and acyl-aminoacids (Supplementary Table S3 < Antismash—novas montagens >). Using the software PRISM 4, we also identified the presence of BGCs encoding ectoine in *Halomonas* (LC_1), *Halopseudomonas* (LC_3), and *Cobetia* (LC_6), and BGCs related to siderophores in *Pseudoalteromonas* (LC_2), *Shewanella* (LC_4) and *Cobetia* (LC_6). Additionally, we annotated non-identified polyketides in all six strains.

Apart from *Pseudoalteromonas* (LC_2), we observed genes related to DMSP metabolism in all genomes (Fig. 3). *Halomonas* (LC_1), *Shewanella* (LC_4), *Brucella* (LC_5), and *Cobetia* (LC_6) presented two copies of the dmdC gene, and *Halopseudomonas* (LC_3) showed six copies. The gene coding 3-methylmercaptopropionyl-CoA dehydrogenase protein is involved in DMSP assimilation^43^. We also found single copies of dddP and dddD in *Cobetia* (LC_6), both of which are related to DMSP lyase protein.

Genes responsible for nitrogen metabolism and regulation of nitrogen fixation were annotated in pSIs of *Halomonas* (LC_1) (Supplementary Table S3, S6). The nitrogen fixation protein FixQ was found in one pSI of *Halopseudomonas* (LC_3); fixK was also detected in *Brucella* (LC_5) (Supplementary Table S6). Nitrification processes were widespread among the genomes. In *Halomonas* (LC_1), nasA (Nitrate reductase), nasD (Nitrite reductase), and nirD (Nitrite reductase) genes were found. We found nrtA (Nitrate transport protein NrtA), nasA, napA (Nitrate reductase), nasD, nirD and narL (Nitrate/nitrite response regulator protein NarL) in *Halopseudomonas* (LC_3). In *Shewanella* (LC_4), napA, napB, nifH (that encodes Nitrogenase iron protein), narL and narQ (Nitrate/nitrite sensor protein NarQ) were detected. The nitrate reductase narGHJI gene was found in *Brucella* (LC_5), as were nirK (Copper-containing nitrite reductase) and nirQ (Denitrification regulatory protein NirQ). The gene for nitrate/nitrite transporter NrtP and the genes napA, nasA, nasD and narQ were observed in *Cobetia* (LC_6). Moreover, in *Halopseudomonas* (LC_3) the gene cobA for uroporphyrinogen-III C-methyltransferase in pSIs was found, which has a key-function in cobalamin (vitamin b12) biosynthesis (Supplementary Table S3).

### Pangenome analyses

**Figure 4:**
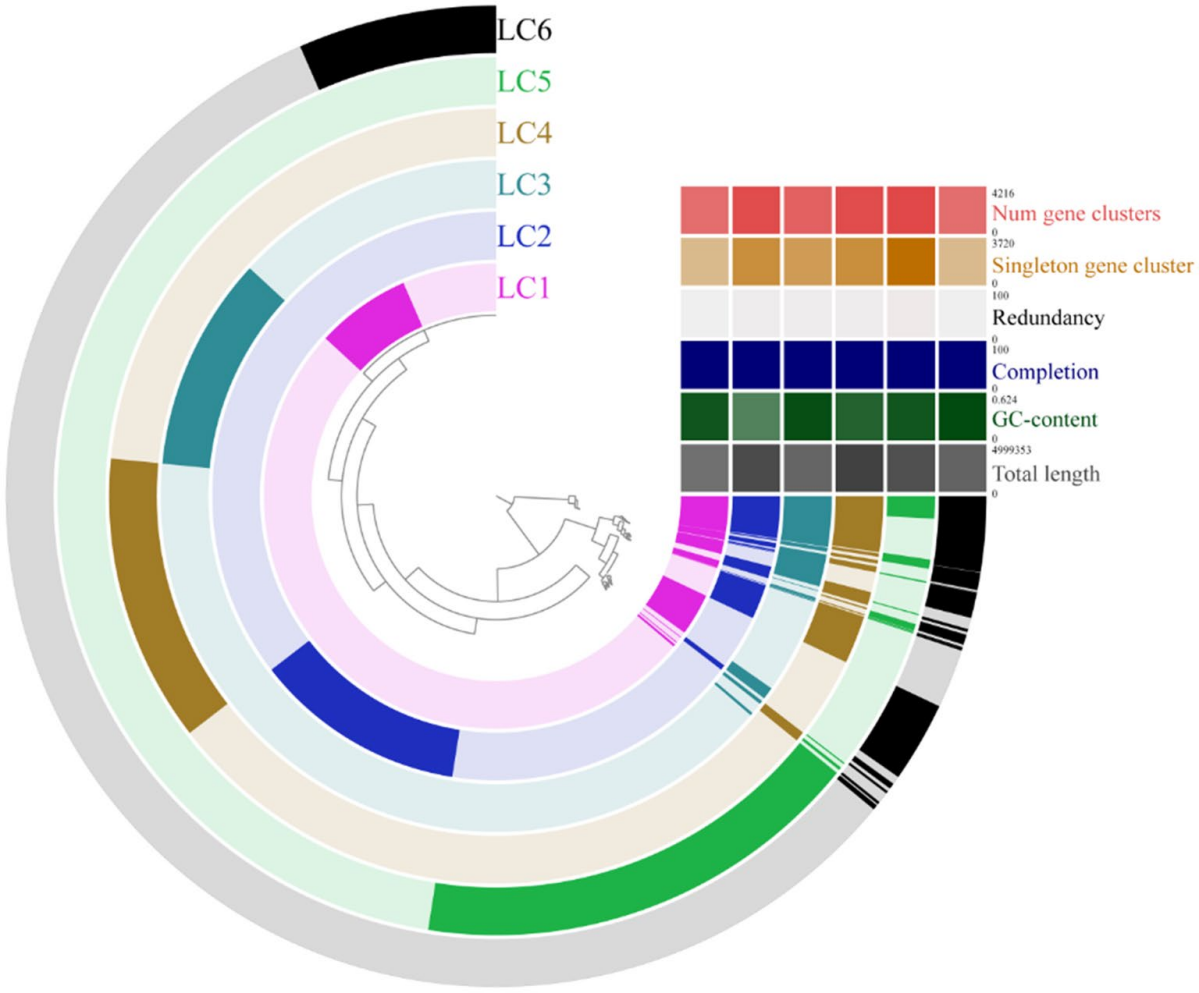
Pangenome analysis comparing the oil-degrading bacteria strains of the consortium. The presence and absence of coding sequences (CDS) in the genome are indicated in dark and light colors, respectively. Basic information (lines) of each genome (columns) is available at the end of the sequences. In the first line (from top to bottom), the number of gene clusters (0–4216) in red, singleton gene clusters (0–3720) in yellow, redundancy (0–100) in light grey, completion (0–100) in blue, GC-content (0–0.624) in green, and total length (0–4,999,353) in dark grey are presented.

The comparative analysis of the members of the consortium indicated both complementary and redundant genes involved in oil degradation (Fig. 4). Although each strain has its own set of unique genes, most genes are shared by at least two strains Supplementary Table S4 and Supplementary Table S5.

**Figure 5:**
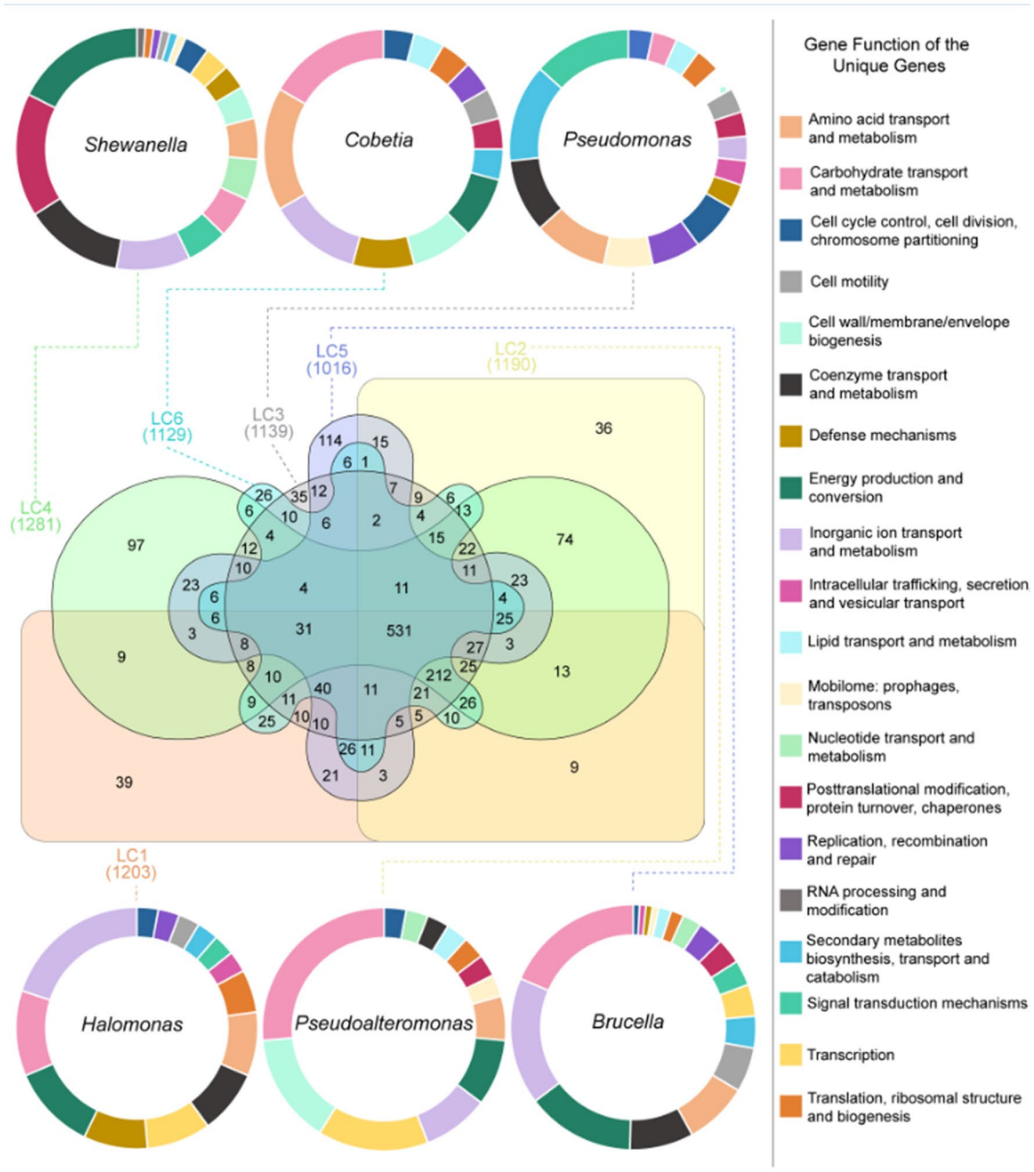
Venn diagram showing the number of shared and unique genes of all the strains. From the pool of unique genes of each strain, it is possible to observe the percentage of genes that perform each function and how this pattern differs among different strains (represented by the individual circles).

Most strains shared a high number of genes with each other, with the exception of Brucella (LC_5) and Shewanella (LC_4), which presented a high number of unique genes (i.e., 114 and 97, respectively; Fig. 5).

## Discussion

Our results support the proposition that genome analysis can be used as a rapid and efficient tool to screen beneficial microorganisms and assemble microbial consortia that can be applied in different situations for marine protection and conservation. The bacterial strains of the genera *Brucella, Cobetia, Halomonas, Halopseudomonas, Pseudoalteromonas,* and *Shewanella* studied here have also been previously reported as ODB in different studies^17,44–46^. Additionally, strains belonging to some of these genera, such as *Pseudoalteromonas*, *Halomonas*, and *Cobetia*, have been applied as coral probiotics, protecting the host against pathogens and heat stress^47^. The investigation of genes related to both mitigation of toxic compounds (oil degradation) and other beneficial traits is key to rapidly understand and optimize our strategies to assemble coral probiotic consortia.

### Mitigation of toxic compounds: the potential of the microbial strains to degrade different oil fractions

A *Halopseudomonas* sp. strain and a *Brucella* sp. strain have been previously assembled together in a successful bioremediation consortium that was able to remove more than 50% of the crude oil from contaminated soil in only seven days^45^. Here, we showed that *Halopseudomonas* (LC_3) and *Brucella* (LC_5) strains have a higher number of genes related to the metabolism of n-alkanes, such as alkane monooxygenases, which are bacterial enzymes related to aerobic degradation of medium‐chain‐length (C5–C11) and long‐chain‐length alkanes (> C12)^48,49^; Fig. 3). AlkT, the gene codifying rubredoxin-NAD( +) reductase, which is another important enzyme for alkane degradation that is involved in the hydrocarbon hydroxylating system^50^, was annotated in *Halopseudomonas* (LC_3), but also in *Halomonas* (LC_1) and *Cobetia* (LC_6). As oil spills release high proportions of n-alkanes in marine environments^48^, the presence of efficient alkane degraders is a key element for an efficient oil-degrading consortium.

The concomitant degradation of aromatic and polyaromatic hydrocarbon (PAH) compounds is crucial for the bioremediation of oil in marine ecosystems. Recent data have shown the bioaccumulation of PAHs in coral tissues^51^, and some of these compounds are known for their high toxicity for these organisms^52^;^53^. Here, we found five genes related to naphthalene degradation among our six ODB genomes (Fig. 2). We also annotated several *phn* genes in *Brucella* (LC_5), which may play an important role in the metabolism of phenanthrenes^54^. Nitrogen cycling also seems to be associated with PAHs’ degradation, as a strong positive correlation between the presence of genes related to nitrate reduction and genes involved in the degradation of PAHs, mainly naphthalene and fluoranthene, has been detected in marine sediments^55^. *Brucella* (LC_5) presents several genes related to respiratory nitrate reductase and denitrification. The correlation between these two processes in marine ecosystems and in the coral tissue needs to be further investigated.

The bphI-bphJ system is responsible for phenol degradation through the breakdown of both 4-hydroxy-2-oxopentanoate and 4-hydroxy-2-oxohexanoate into pyruvate, releasing aldehyde residues. Bphl catalyzes the cleavage of both molecules, while BphJ has dehydrogenase action on potentially toxic aldehyde residues^56,57^. Our consortium showed the potential to degrade 4-hydroxy-2-oxopentanoate and 4-hydroxy-2-oxohexanoate, as bphI and bphJ genes were found in *Halopseudomonas* (LC_3), and bphl was detected in *Pseudoalteromonas* (LC_2). Moreover, genes encoding key components for toluene and xylene degradation were observed in *Halomonas* (LC_1), *Pseudoalteromonas* (LC_2), *Halopseudomonas* (LC_3), and *Cobetia* (LC_6). *Halopseudomonas* (LC_3) showed several toluene and xylene degradation-related genes. The genus *Halopseudomonas* is known for being able to degrade these compounds^58,59^.

Even though *Shewanella* (LC_4) genome analysis revealed only one gene related to hydrocarbon degradation, this strain was still able to grow on the media containing crude oil as the only carbon source. It is possible that *Shewanella* (LC_4) took advantage of the oil degradation arsenal of the other isolates, using subproducts of their metabolisms or even consuming the agar from the solid medium. *Shewanella* strains are known for their versatility to grow under different and adverse conditions because of their ability to use a wide range of electron acceptors and substrates^60–62^. Additionally, members of this microbial genera have been reported as symbionts of several marine hosts, being applied as probiotics in fish farming^63^ and for the bioremediation of several toxic compounds^61^. Thus, this strain may be playing a crucial role in promoting coral health by both degrading toxic compounds and through additional symbiotic interactions.

Although not all strains presented several genes or complete pathways for hydrocarbon degradation, when combined, the six ODB strains showed the potential to perform different steps involved in oil-degradation pathways (Fig. 2). We, therefore, reinforce the importance of using complementary microbial strains as a consortium, instead of a single strain, for oil bioremediation efforts.

### The oil-degrading consortium harbors genes related to coral-microbiome interaction and beneficial traits to the host

Coral-associated microbes contribute to the growth^64^, health, and resilience of the host, playing essential functional roles, such as protection against pathogens^47,65–69^, stress response^47,70^, nutrient acquisition^71,72^, and nitrogen cycling^73–75^, in addition to the mitigation of toxic compounds^1,2^. The manipulation of these beneficial traits has been proposed as a tool to increase corals’ capacity to adapt to environmental changes^23,24,76,77^ and minimize biodiversity loss^5^. In this study, we detected several putatively beneficial genes for corals in the genomes of ODB, such as genes related to bacterial secretion systems, motility, sporulation, nitrogen metabolism and fixation, DMSP metabolism, cobalamin biosynthesis, and the production of antimicrobial compounds and cell protectants.

Bacterial secretion systems may be important for coral-associated bacteria to interact with their hosts. It has been observed enrichment of secretion systems, types II and IV, in bacteria in the coral ecosphere (i.e., seawater environment surrounding coral)^78^. Symbionts can use these systems not only to deliver and secrete molecules but also to establish on surfaces. Additionally, it can foster endocytosis of the cell host, among other functions related to microbial-host interactions^79^. The pSIs identified in *Halomonas* (LC_1) and *Cobetia* (LC_6) genomes were rich in TRAP and ABC transporters, which are also important for microbial-host interactions^80,81^. Flagellar motility and chemotaxis are also essential for bacterial symbionts to reach and colonize the coral host^82–84^. Genes related to motility and chemotaxis were also annotated in our ODB genomes.

The production of antimicrobial compounds, which has been widely reported in coral-associated bacteria^66,85–87^, can drive the protection of the host against pathogens while modulating the coral microbiome^85,88–90^. RiPP-like BGCs, antimicrobial candidates, were annotated in *Pseudoalteromonas* (LC_2), *Halopseudomonas* (LC_3), *Shewanella* (LC_4) and *Cobetia* (LC_6; Supplementary Table S3 < Antismash—novas montagens >). We also observed T1PKS in *Halomonas* (LC_1) and *Cobetia* (LC_6), a compound known to exhibit broad antimicrobial activities^91,92^.

Ectoin and arylpolyene related-BGCs are known to protect cells against oxidative effects^93,94^. We identified ectoin BGCs in *Halomonas* (LC_1), *Halopseudomonas* (LC_3) and *Cobetia* (LC_6), while arylpolyene BGCs were found in *Pseudoalteromonas* (LC_2), *Shewanella* (LC_4), *Brucella* (LC_5) and *Cobetia* (LC_6). Genes that encode ectoine TRAP transporters, teaABCD, were also found in *Halomonas* (LC_1). We also found teaA and teaC genes in *Halopseudomonas* (LC_3) and in *Cobetia* (LC_6) genomes. In addition, uehB, which encodes Ectoine/5-hydroxyectoine TRAP transporter small permease protein UehB, was annotated in *Halopseudomonas* (LC_3). These results reinforce the production of ectoine by these strains and also its role as a BMC mechanism^88^.

DMSP is central in the marine sulfur cycle and naturally produced by the coral dinoflagellate symbionts^95^, frequently used as a source of sulfur by different members of the holobiont. However, excessive amounts of DMSP can also act as a cue for opportunistic and pathogenic bacterial chemotaxis^96^, and may trigger dysbiotic processes. Therefore, mainly through demethylation and cleavage, the degradation of DMSP can avoid the colonization of potential pathogenic bacteria while providing additional sources of carbon and reduced sulfur. The gene dmdC, found in *Halomonas* (LC_1), *Shewanella* (LC_4), *Brucella* (LC_5) and *Cobetia* (LC_6), encodes for the enzyme 3-methylmercaptopropionyl-CoA (MMPA-CoA) dehydrogenase. This enzyme catalyzes the synthesis of methylthioacrylyl from 3-methiolpropionate, in a key reaction in the demethylation pathway^97^. However, other components are required for the complete demethylation pathway, including dmdA, dmdB and dmdC^43^. We also observed dddD and dddP, both coding DMSP lyase proteins, in the *Cobetia* (LC_6) genome. This enzyme is the unique component responsible for the cleavage pathway of DMSP into DMS + acrylate^97^. We annotated components of the DMSP assimilation pathways in *Halomonas* (LC_1) and *Cobetia* (LC_6). The genera *Halomonas* and *Pseudoalteromonas* have been previously reported to be involved in the metabolism of DMSP^98^. Members of the genus *Pseudomonas* have already been observed to use DMSP as the only carbon source^99^, while members of the genus *Shewanella* have been previously characterized by their ability to metabolize DMSP into DMS^100,101^. However, here we report the first annotated DMSP assimilation pathways in a member of the *Brucella* genus (LC_5).

Dinoflagellate symbionts and coral cells have a deficiency in biosynthesis of cobalamin, and evidence showed that gastric-cavity-associated microbes can supply this vitamin for both coral cells and Symbiodiniaceae^102–104^. Uroporphyrinogen III is a key intermediate to cobalamin biosynthesis^105^. This vitamin is important for several functions, including amino acids biosynthesis. We found two copies of a gene that encodes uroporphyrin-III C-methyltransferase, a protein related to the conversion of uroporphyrinogen III to precorrin-2, in different pSIs of *Halopseudomonas* (LC_3). Vitamin production genes have been previously annotated in members of a bacterial consortium that protected coral health against heat stress and pathogens^88^.

Nitrogen cycling in corals is performed by different members of the holobiont, and nitrogen-cycling microbes are consistently found across the microbiomes of different species of reef-building corals^74^. Indeed, corals rely on the fine balance between nitrogen availability and the presence of excessive amounts of ammonia, which has been recently proposed as one of the main causes of coral bleaching^75^. Thus, having symbionts that play roles in different pathways related to nitrogen cycling, such as nitrogen fixation, denitrification, and nitrification, is extremely important to maintain a healthy holobiont. Nitrification is thought to prevent ammonium loss, retaining the nitrogen present in the molecule^74,106^. On the other hand, N-fixation works as a direct way to deliver nitrogen for both dinoflagellates and host cells^73,107^. We found several genes involved in nitrification processes in *Halopseudomonas* (LC_3)*, Shewanella* (LC_4)*,* and *Cobetia* (LC_6), and genes related to N-fixation *Brucella* (LC_5) and *Cobetia* (LC_6), indicating the potential of this bacterial consortium to be contributing to the regulation and functioning of nitrogen cycling within the coral holobiont (Supplementary Table S6). On the other hand, *Brucella* (LC_5) showed genes related to nitrate reduction to nitrite and gene for nitrite reduction to nitric oxide, indicating it role in denitrification rather than nitrification (Supplementary Table S6). Thus, nitrification, denitrification and N-fixation are potential traits for the consortium.

**Figure 6:**
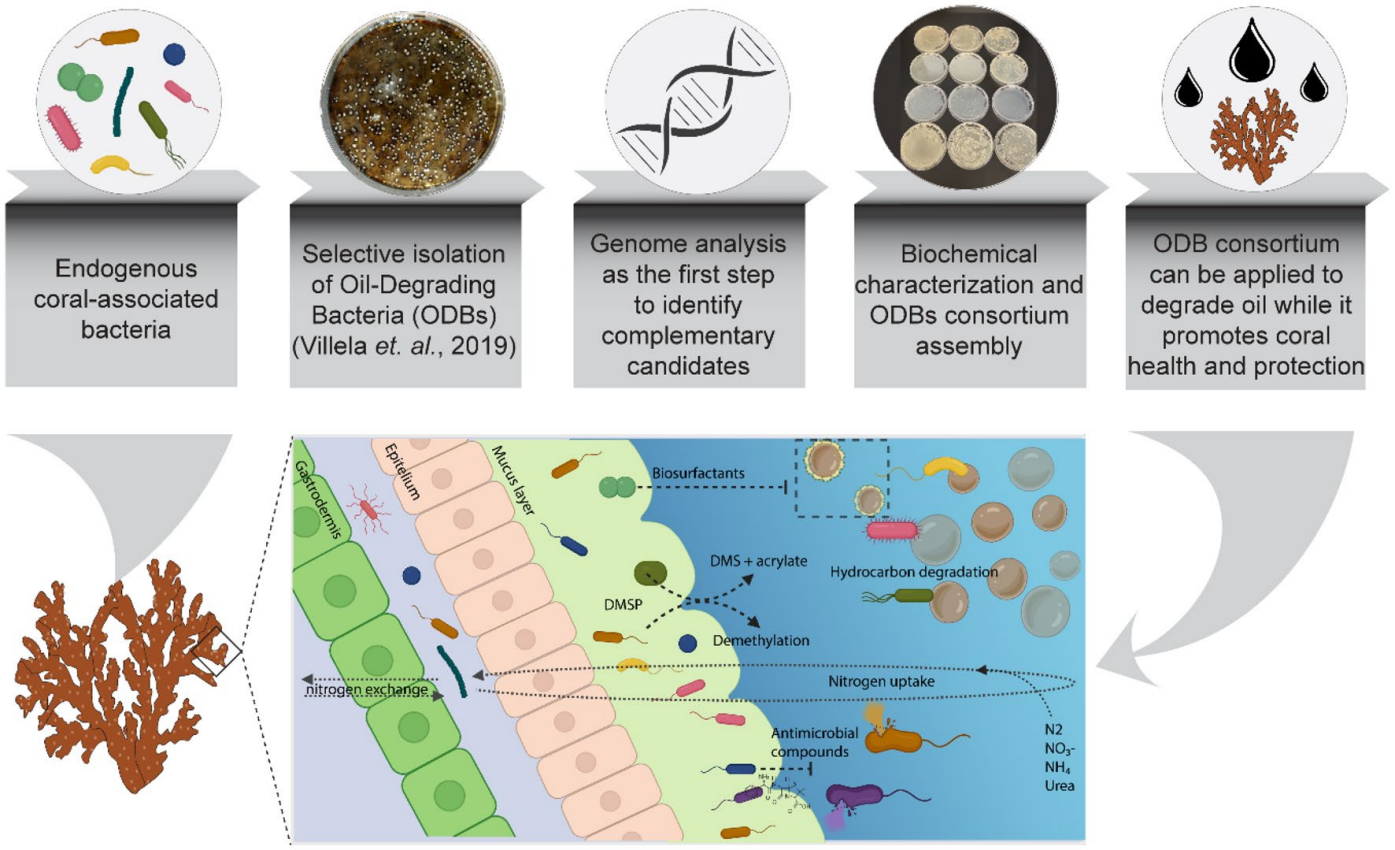
A framework to isolate, test, and re-apply a complementary beneficial microorganism for corals-oil degrading bacteria consortium. ODB are naturally found associated with corals and reef waters. These bacteria can be selectively isolated using specific media. Genome analysis can be the first step for a consortium assembly because it allows the selection of individual isolates able to degrade different oil fractions and promote coral health (BMC traits). After this step, biochemical tests can be performed to confirm the ability of degrading oil, among other potential beneficial characteristics. In the end, the consortium is ready to be tested to degrade oil, while it can also offer many other beneficial traits to the host.

Our results highlight the potential of ODB to promote the concomitant bioremediation of oil and increase in the overall coral health status via other microbial-mediated mechanisms. A summary of a protocol to isolate, test and grow a complementary BMC-ODB consortium from coral reef ecosystems, as well as the consortium’s proposed mechanisms to mitigate toxic compounds and promote benefits to coral health is shown in Fig. 6.

## Conclusions

Our findings highlight the importance of studying the genomes of ODBs to customize a complementary and efficient BMC-ODB consortium^76^. These ODBs were part of a consortium that was able to mitigate the impacts of hydrocarbon contamination on corals and significantly degrade PAHs and N-alkanes^2^. We provide the genetic basis that explains the importance of using a BMC consortium (and not single strains) to maximize the bioremediation potential of microbes, as they show the ability to degrade different fractions of oil, in a complementary way. Furthermore, the present approach allowed the identification of various additional beneficial mechanisms^108^ offered by the selected consortium that may mitigate the cascade of coral disorders triggered by oil-driven dysbiosis. We propose a framework based on genome analysis to select a complementary BMC-ODB consortium that can increase oil biodegradation while also promoting additional benefits to coral health.

## Supplementary Information

Supplementary Figure 1.

Supplementary Legends.

Supplementary Table S1.

Supplementary Table S2.

Supplementary Table S3.

Supplementary Table S4.

Supplementary Table S5.

Supplementary Table S6.

## Data Availability

All genome sequences were deposited in GenBank under the accessions SAMN34403400 (LC1), SAMN34405137 (LC2), SAMN34405138 (LC3), SAMN34405139 (LC4), SAMN34405140 (LC5), SAMN34405141 (LC6), in the BioProject PRJNA962487.
