# Supplementary figures and images for: Genome analysis of a coral-associated bacterial consortium highlights complementary hydrocarbon degradation ability and other beneficial mechanisms for the host
Source: Sci Rep. 2023 Jul 28;13:12273. doi: 10.1038/s41598-023-38512-z (PMC10382565; doi:10.1038/s41598-023-38512-z)

Tree scale: 0.1

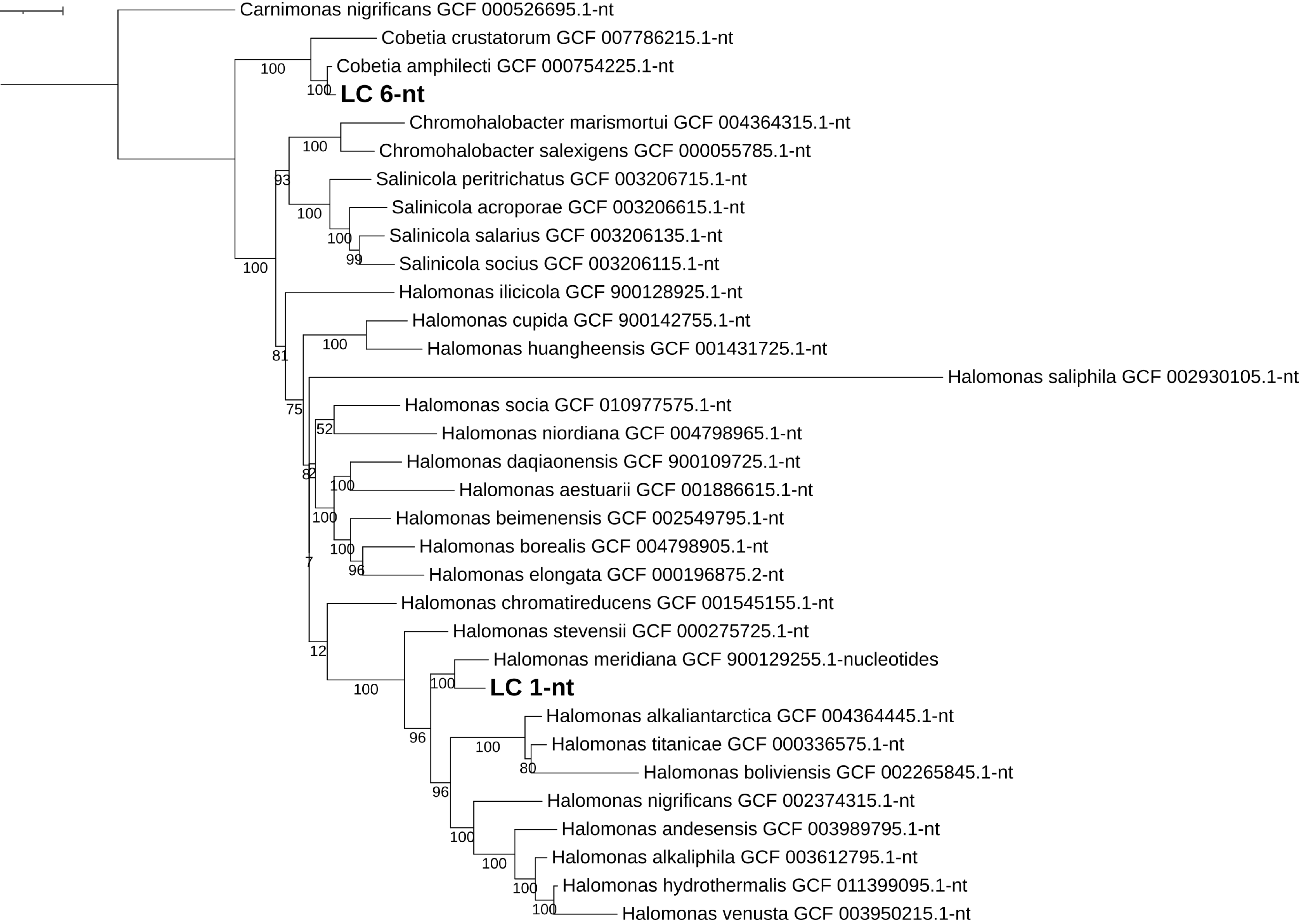

Tree scale: 0.1

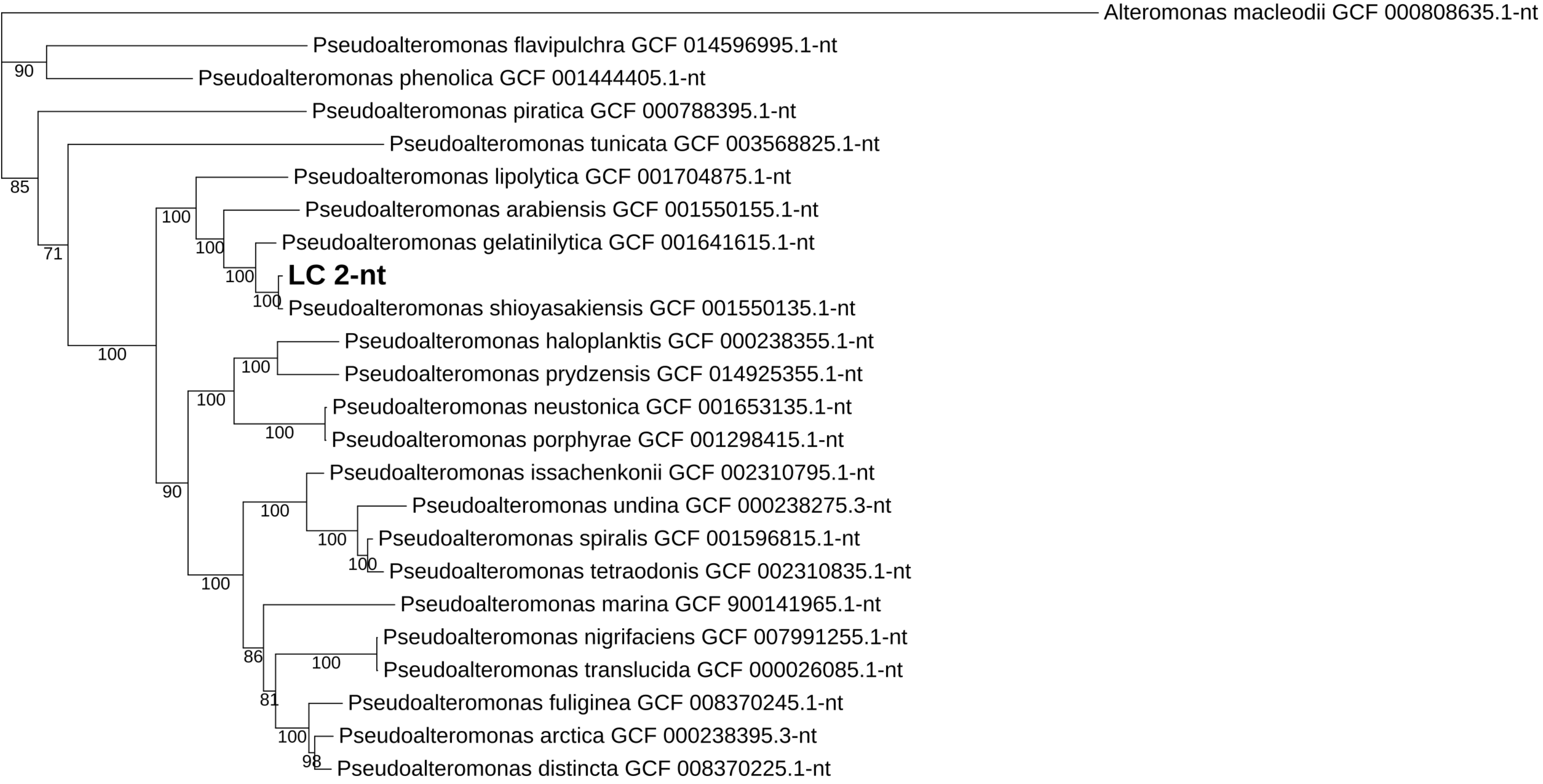

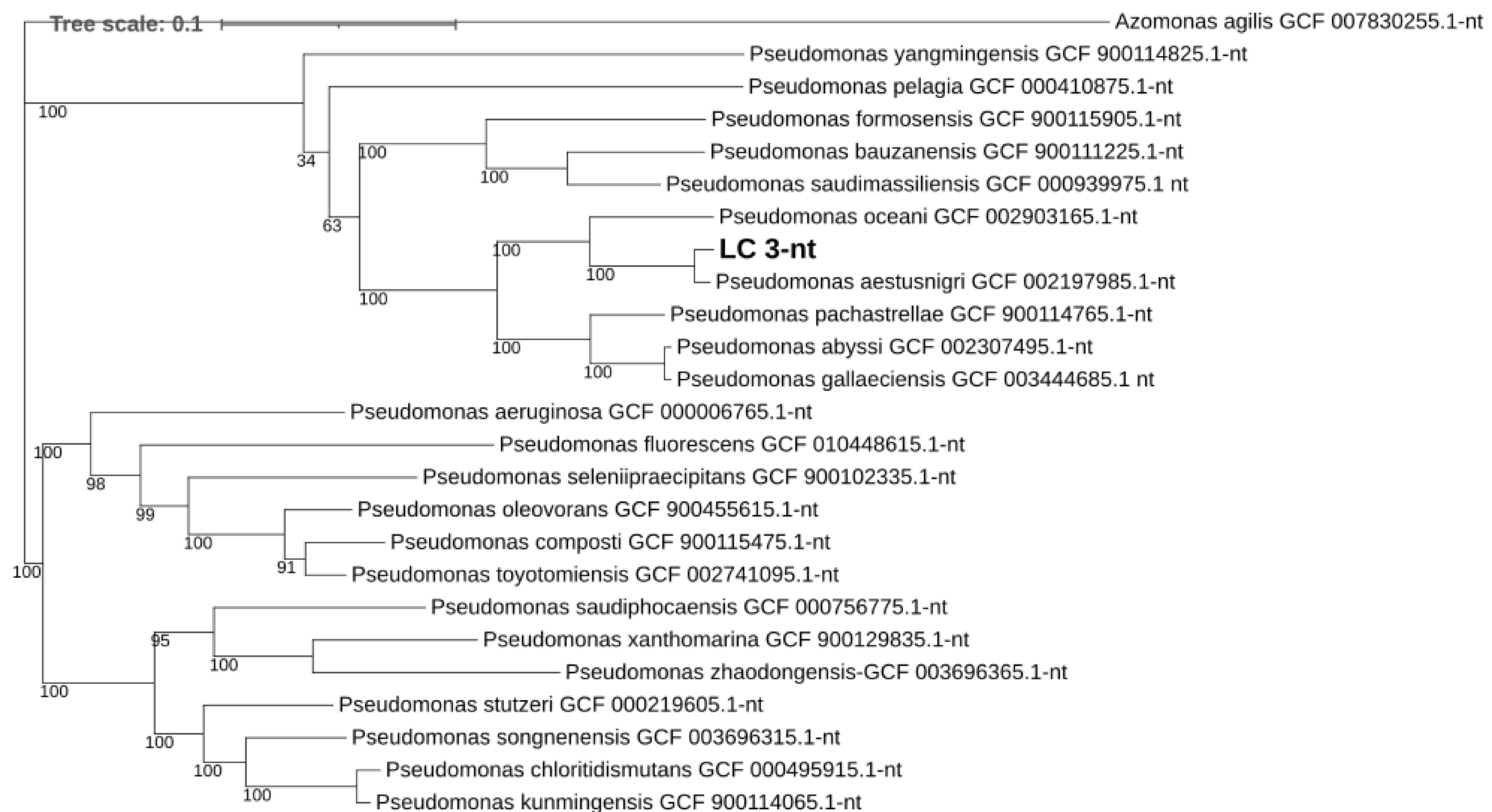

Tree scale: 0.1

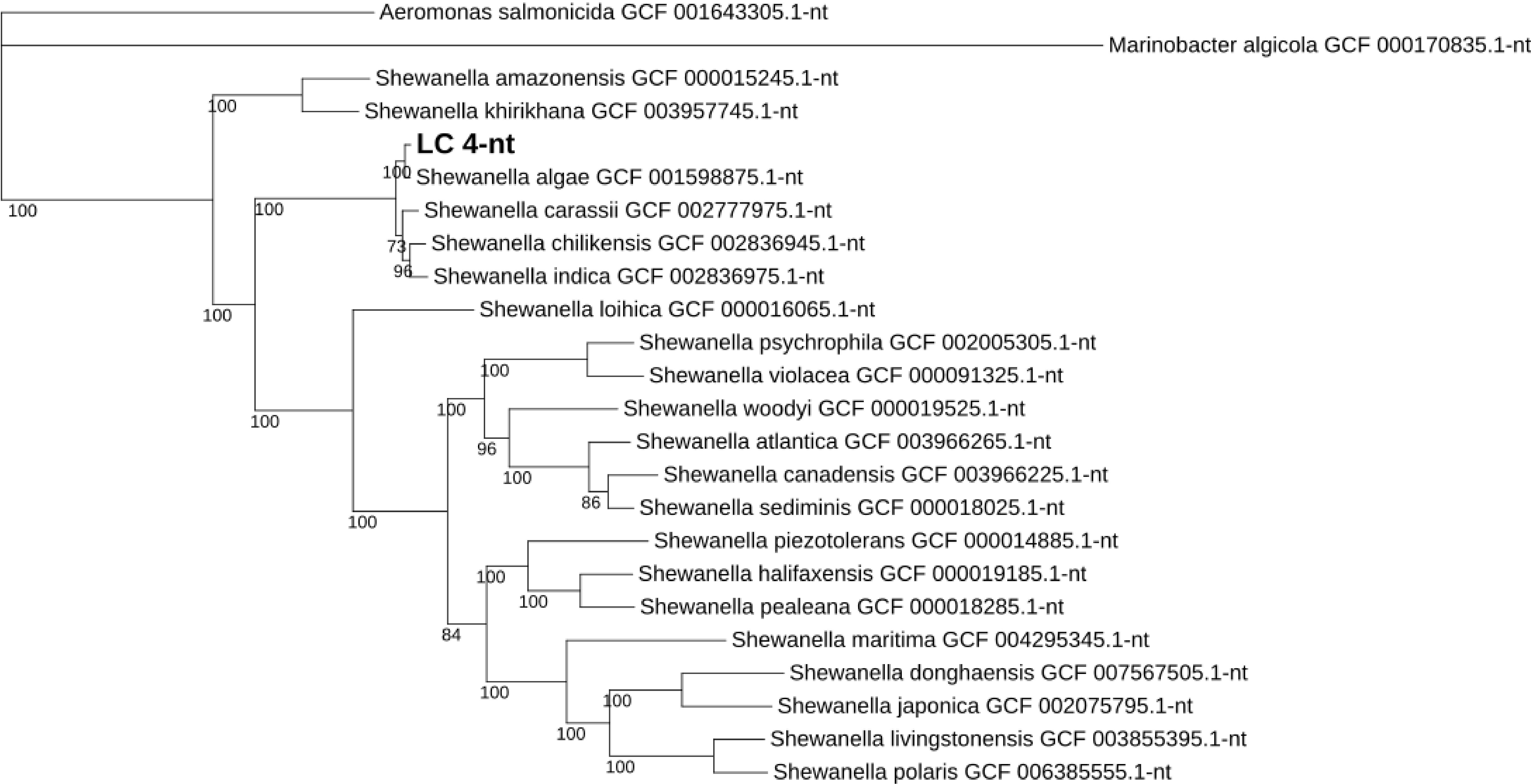

Tree scale: 0.1

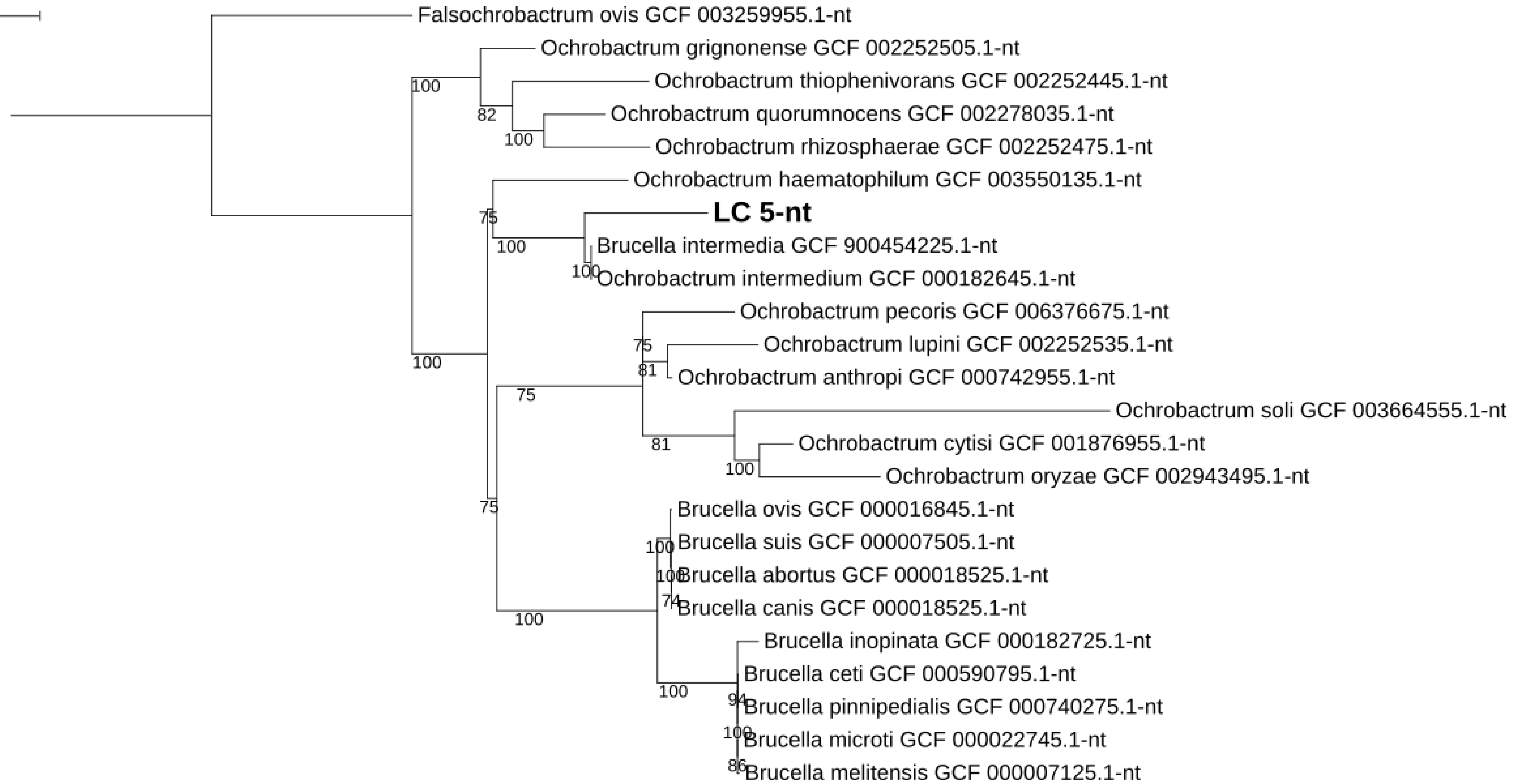

Supplement: Supplementary file 1 — Supplementary Figure 1. [file 41598_2023_38512_MOESM1_ESM.pdf]
