# Supplementary material for: Genome analysis of a coral-associated bacterial consortium highlights complementary hydrocarbon degradation ability and other beneficial mechanisms for the host
Source: Sci Rep. 2023 Jul 28;13:12273. doi: 10.1038/s41598-023-38512-z (PMC10382565; doi:10.1038/s41598-023-38512-z)
Supplement: Supplementary file 2 — Supplementary Legends. [file 41598_2023_38512_MOESM2_ESM.docx]

Supplementary Figure 1. Phylogenetic trees showing the taxonomic identification of the ODB strains.

Supplementary Table S1. List of marker genes used for Multilocus Sequence Analysis of ODB strains.

Supplementary Table S2. Genes from symbiotic islands predicted in GIPSy.

Supplementary Table S3. Biosynthetic gene clusters predicted in AntiSMASH 5.0.

Supplementary Table S4. Unique genes of each strain annotated in the Pangenome analysis and their functions.

Supplementary Table S5. Pangenome analysis output (unique and shared genes).
